# Guidelines for reporting methods to estimate metabolic rates by aquatic intermittent-flow respirometry

**DOI:** 10.1242/jeb.242522

**Published:** 2021-09-14

**Authors:** Shaun S. Killen, Emil A. F. Christensen, Daphne Cortese, Libor Závorka, Tommy Norin, Lucy Cotgrove, Amélie Crespel, Amelia Munson, Julie J. H. Nati, Magdalene Papatheodoulou, David J. McKenzie

**Affiliations:** 1 Institute of Biodiversity, Animal Health and Comparative Medicine, College of Medical, Veterinary and Life Sciences, Graham Kerr Building, University of Glasgow, Glasgow G12 8QQ, UK; 2 PSL Université Paris: EPHE-UPVD-CNRS, USR 3278 CRIOBE, BP 1013, 98729 Papetoai, Moorea, French Polynesia; 3 WasserCluster Lunz–Inter-university Centre for Aquatic Ecosystem Research, A-3293 Lunz am See, Austria; 4 DTU Aqua: National Institute of Aquatic Resources, Technical University of Denmark, 2800 Kgs. Lyngby, Denmark; 5 Department of Biology, University of Turku, 20500 Turku, Finland; 6 Department of Environmental Science and Policy, University of California, Davis, CA 95615, USA; 7 MARBEC, Université Montpellier, CNRS, Ifremer, IRD, 34000 Montpellier, France; 8 Enalia Physis Environmental Research Centre (ENALIA), 2101 Nicosia, Cyprus

**Keywords:** Metabolic rate, Fish, Oxygen, Aerobic metabolism, Replication, Experimental design

## Abstract

Interest in the measurement of metabolic rates is growing rapidly, because of the importance of metabolism in advancing our understanding of organismal physiology, behaviour, evolution and responses to environmental change. The study of metabolism in aquatic animals is undergoing an especially pronounced expansion, with more researchers utilising intermittent-flow respirometry as a research tool than ever before. Aquatic respirometry measures the rate of oxygen uptake as a proxy for metabolic rate, and the intermittent-flow technique has numerous strengths for use with aquatic animals, allowing metabolic rate to be repeatedly estimated on individual animals over several hours or days and during exposure to various conditions or stimuli. There are, however, no published guidelines for the reporting of methodological details when using this method. Here, we provide the first guidelines for reporting intermittent-flow respirometry methods, in the form of a checklist of criteria that we consider to be the minimum required for the interpretation, evaluation and replication of experiments using intermittent-flow respirometry. Furthermore, using a survey of the existing literature, we show that there has been incomplete and inconsistent reporting of methods for intermittent-flow respirometry over the past few decades. Use of the provided checklist of required criteria by researchers when publishing their work should increase consistency of the reporting of methods for studies that use intermittent-flow respirometry. With the steep increase in studies using intermittent-flow respirometry, now is the ideal time to standardise reporting of methods, so that – in the future – data can be properly assessed by other scientists and conservationists.

## Abstract (precis)

**Summary:** The first checklist of guidelines for reporting methods of intermittent-flow respirometry to enhance study replicability. Methods reporting for this technique has been inconsistent and incomplete in peer-reviewed articles.

## Introduction

Estimation of the metabolic rates of animals has been a core element of research in comparative physiology for decades (Kleiber, 1947; Rolfe and Brown, 1997). Metabolic rates have also been studied in the context of physiological and behavioural ecology (Killen et al., 2013; Mathot et al., 2019; Metcalfe et al., 2016), as well as in the examination of broad ecological phenomena across levels of biological organisation (Brown et al., 2004; Hatton et al., 2019). The study of metabolic rates has recently received even greater attention because of the need to understand plastic and evolutionary responses to environmental change, particularly in aquatic ecosystems (Jutfelt et al., 2018; Norin and Metcalfe, 2019; Pörtner et al., 2017). This increased interest has occurred alongside technological advances in methods of respirometry, which measure rates of gas exchange between an organism and their environment. In particular, the rate at which an organism takes up oxygen from its environment is expected to be related stoichiometrically to rates of ATP production by mitochondrial oxidative phosphorylation and, therefore, is considered a proxy for metabolic rate (Nelson, 2016). The rise of commercially available components has further facilitated the estimation of metabolic rates by respirometry in a variety of organisms. These factors have been particularly consequential for respirometry on animals that breathe water because, historically, this has been more difficult to conduct compared with respirometry on air-breathers. As such, there are more scientists using aquatic respirometry as a research tool than ever before, with more than 60% of the papers in this field being generated in the past 10 years alone (Fig. S1).

We begin this Commentary by describing methods of aquatic respirometry, particularly focusing on intermittent-flow respirometry, and go on to discuss the need to standardise the reporting of methods for studies using this research technique. We then provide a checklist of 53 essential methodological criteria that should be reported in all studies using intermittent-flow respirometry. We also present results of a literature survey, demonstrating the extent to which these various criteria have traditionally been inadequately reported. Finally, we provide a downloadable form (Table S1) that we encourage researchers to complete and include with future manuscripts for studies using intermittent-flow respirometry, to clearly and concisely summarise key methodological details.

Box 1. Metabolic traits that can be estimated using intermittent-flow respirometry **Standard metabolic rate (SMR)**

This is the minimum rate of ATP use required to sustain life, in the absence of voluntary muscular movements and digestion/absorption of nutrients (Chabot et al., 2016). With intermittent-flow respirometry, SMR is estimated by collecting measurements of oxygen uptake over an extended period on an undisturbed animal, after acclimatation to the respirometer, and then extracting a value for SMR using one of a number of statistical methods (Chabot et al., 2016). In ectotherms, SMR is especially likely to change with environmental temperature (Chabot et al., 2016; Schulte, 2015), so temperature must be reported.

**Routine metabolic rate (RMR)**

This is the average oxygen uptake rate of a post-absorptive animal, where spontaneous activity contributes to ATP use and, therefore, oxygen demand (Chabot et al., 2016). It is typically measured as the average of the oxygen uptake rate measurements that are collected to estimate SMR, although some portions of the dataset may not be considered; for example, high rates of oxygen uptake when the animal is stressed by handling for placement in the respirometer (Chabot et al., 2016; Steffensen, 1989). The RMR can, in theory, lie anywhere between SMR and MMR, but it is expected to be closer to SMR if animals are undisturbed (Chabot et al., 2016). RMR is often used to infer a metabolic response to a stimulus or stressor (e.g. perceived predator threat) (Hall and Clark, 2016; Palacios et al., 2016). Also note that the abbreviation ‘RMR’ is sometimes used to refer to ‘resting metabolic rate’, a term often used as a less strict equivalent of SMR.

**Maximum metabolic rate (MMR)**

This is the maximum rate of oxygen uptake that an animal can achieve to create ATP aerobically (Norin and Clark, 2016). Two main methods are used to estimate MMR in fishes (Killen et al., 2017; Norin and Clark, 2016): they can be exposed to incremental swim speeds in a swim-tunnel respirometer, with MMR taken as the highest rate of oxygen uptake before fatigue, or they can be chased to exhaustion in a tank and then placed in a respirometer chamber, with MMR taken as the highest rate of oxygen uptake during recovery. There is no consensus on how best to measure MMR (Killen et al., 2017; Norin and Clark, 2016; Zhang et al., 2020).

**Aerobic metabolic scope (AS)**

This is the maximum capacity to supply oxygen to sustain metabolic activities beyond SMR (Fry, 1971). Absolute AS is calculated as MMR minus SMR, whereas factorial AS is MMR divided by SMR; the choice of which is more appropriate may depend on the research question of interest (Halsey et al., 2018).

## Intermittent-flow respirometry

**Fig. 1.**
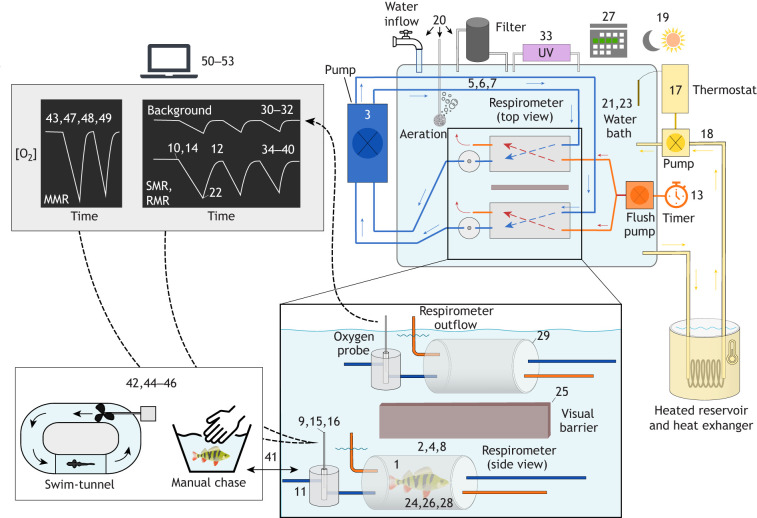
**Schematic of a typical intermittent-flow respirometry setup.** Numbers correspond to the criteria listed in Table 1 and show the general location of each criterion within the setup. The top right depicts a top-down view of the setup; the enlarged box below presents a more detailed side-view of two respirometers (one containing a fish and the other being an empty chamber for measuring background respiration). Orange items (excluding the sun in the top corner, which represents photoperiod) are those used for periodically flushing the respirometer with clean, aerated water from the surrounding bath, with orange lines representing tubing in this flushing circuit. Dark blue represents the mixing circuit and associated tubing. Note that in this scheme, mixing is performed with a multi-channel peristaltic pump, but mixing can also be achieved with a single-channel pump or stir-bar, depending on the size and shape of the respirometers. Yellow represents elements associated with temperature control; here, temperature is maintained using a thermostat that controls a pump to direct water through a heat exchanger within a heated reservoir whenever temperature within the bath drops below the setpoint. The box to the lower left depicts methods for exercising fish for estimates of maximum metabolic rate. The top left box represents computer-based data collection and analyses. Dashed black arrows represent transmission of data from oxygen probes to computer for analyses. Refer to Svendsen et al. (2016a,b) for more information on setup components and overall system functioning. SMR, standard metabolic rate; RMR, routine metabolic rate; MMR, maximum metabolic rate; UV, ultraviolet.

The most widely accepted method for measuring rates of oxygen uptake in water-breathing organisms is automated intermittent-flow respirometry (Steffensen, 1989; Svendsen et al., 2016a), also sometimes referred to as intermittent-closed respirometry (Norin and Gamperl, 2018). Although the technique has mainly been developed for use on fishes, it is suitable for almost any water-breathing organism (Fig. 1). An animal is placed in a gas-impermeable respirometry chamber equipped with an oxygen sensor, and is then exposed to periodic, alternating ‘closed’ and ‘flush’ phases. During the closed phase, the respirometer is effectively sealed and there is a decline in dissolved oxygen in the water due to oxygen uptake by the animal. With traditional closed respirometry, the decline in oxygen concentration is measured while the animal is in a continually sealed chamber, and this containment can eventually cause hypoxia and accumulation of waste products in the respirometer, which can influence the resulting measurements of oxygen uptake. With intermittent-flow respirometry, however, this is avoided by the flush phase, during which the respirometer is flushed with clean, aerated water, which replaces oxygen and removes metabolic wastes.

**Table 1.**
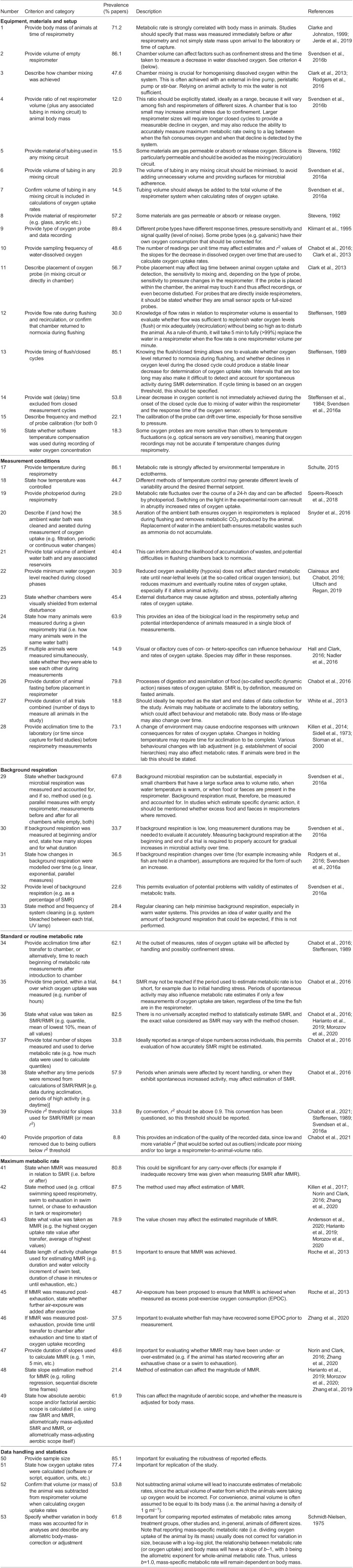
Checklist of criteria that should be reported when using aquatic intermittent-flow respirometry to estimate SMR/RMR or MMR, along with detailed descriptions of each criterion. Also shown is the prevalence of each criterion in the existing literature

The alternation of the closed and flush phases means that real-time rates of oxygen uptake can be recorded in successive closed phases over extended periods, with animals left undisturbed. This can provide an accurate picture of dynamic changes in oxygen uptake over time. These changes might be due to factors such as initial handling stress, circadian rhythms and metabolic costs of digestion, among others (Jourdan-Pineau et al., 2010; Steffensen, 1989). Using this technique can also reveal when the undisturbed animal is potentially functioning at basal rates of metabolism, denoted as standard metabolic rate (SMR) for ectotherms (Box 1) or when it is performing some defined level of activity or type of behaviour, often referred to as routine metabolic rate (RMR, Box 1). This ability to track changes in oxygen uptake rate in real time is a major improvement over the technique of flow-through respirometry (Ultsch et al., 1980), where there is a continuous flow of water through a respirometry chamber and oxygen uptake is measured by comparing the difference in oxygen concentration at inflow and outflow. This can result in large measurement error when the difference in dissolved oxygen at the inflow and outflow are small. Furthermore, with flow-through respirometry, changes in oxygen concentration at the outflow lag behind changes in metabolic activity of the animal because of a reservoir, or wash-out, effect. This lag depends upon the dilution factor, which is the ratio of respirometer volume to rate of water flow through it (see Steffensen, 1989 for a detailed explanation). The consequence is that flow-through respirometry can only be used to measure relatively steady physiological states (Steffensen, 1989).

Intermittent-flow respirometry is, therefore, the best available method to estimate metabolic rate in water-breathing animals and should be utilised whenever possible. Notably, measures of SMR and RMR can be coupled with measures of aerobic maximum metabolic rate (MMR, Box 1) to estimate aerobic scope (AS, Box 1). Given this wide range of applications and the relative robustness of intermittent-flow respirometry, it has become an extremely popular choice of methodology among comparative physiologists, including researchers who are new to the estimation of metabolic rates via measurement of respiratory gas exchange.

## A need for the standardisation of methods

Despite its increasingly wide usage, there are no guidelines for reporting the methods used in intermittent-flow respirometry. There are several guides to best practice for measuring and analysing various types of metabolic rates (Chabot et al., 2016; Norin and Clark, 2016; Steffensen, 1989; Svendsen et al., 2016a; Clark et al., 2013), but details can vary widely among researchers, and the quality and values of the data generated using intermittent-flow respirometry is completely dependent on myriad methodological decisions made throughout the experiment, from equipment setup to data collection and analysis (Steffensen, 1989; Svendsen et al., 2016a). Equally important is that the reporting of methods also differs greatly across peer-reviewed studies, with important details often not mentioned. A lack of methodological detail, or inaccurate and vague descriptions, are problematic because: (1) they make it difficult for readers to evaluate data reliability and judge the interpretation of results; (2) they can give a misleading impression of what was done; (3) they hinder replication of the experiments; and (4) data on metabolic rates are increasingly used in meta-analyses (Holtmann et al., 2017; Jerde et al., 2019; Killen et al., 2016), so proper methodological documentation would be useful to allow researchers to understand sources of residual variation across studies. In addition, a standard set of guidelines for reporting methods in intermittent-flow respirometry studies would make it easier for journal editors and reviewers to decide whether a given study warrants publication in the first place. Finally, a list of important methodological details would be extremely useful for students and researchers who are new to this field of research and are using this technique for the first time.

Thus, we believe that researchers using intermittent-flow respirometry would benefit from a standardised, publicly available checklist of details that should be included when describing their methods, to prevent further under-reporting of important elements. The use of reporting guidelines for methods, in the form of checklists or flow-charts (Carp, 2012; Cowger et al., 2020; Michel et al., 2020), is widespread across the biological sciences, and is long overdue in comparative physiology and especially respirometry. With the steady increase in the number of published studies using respirometry (Fig. S1), now is the ideal time to establish and institute such standard guidelines for accurate methodological reporting.

## A checklist for reporting methods using intermittent-flow respirometry

Focusing on studies with fishes, here we provide a checklist of 53 criteria that are essential for understanding, interpreting and replicating experiments using aquatic intermittent-flow respirometry (Table 1; Fig. 1). We aim to provide an explicit list of details that can be referenced when writing or evaluating research papers, or when planning new studies. Although the criteria are focussed on studies with fishes, most criteria could be applied to studies with any aquatic organism. In addition to being included as Table 1 of this article, we also provide a downloadable form that authors can use to easily list each criterion in a table format, which we suggest can be submitted as supplementary material with manuscripts to accompany contextual descriptions in the main text of published research papers (Table S1).

We have restricted our criteria to cover methods involved in the most common forms of aquatic intermittent-flow respirometry, namely, the measurement of SMR, RMR, MMR and aerobic metabolic scope (AS) (Box 1). Methods unique to other applications, such as protocols for measuring critical oxygen tension (*P*_crit_), are not specifically covered here (Claireaux and Chabot, 2016; Ultsch and Regan, 2019), but the checklist can still be used as a guide to ensure that the most basic criteria of respirometry are met when carrying out these more specialised procedures.

The criteria are divided into six categories, based on whether they describe the materials and conditions used in any given study, or are details of the various measurements that can be conducted using intermittent-flow respirometry. More detail on each of the categories in the checklist is given below.

### 

#### Equipment, materials and setup

It is necessary to provide adequate details of the specific equipment used in the study and the way these components are assembled to measure animal oxygen uptake. Equipment and setup details are important because there is a wide array of available oxygen sensors, data acquisition devices and logging software, as well as different options for respirometer construction and virtually every other component used in intermittent-flow respirometry. Each choice made when gathering equipment and setting up the apparatus can potentially have an impact on the results obtained. Consequently, depending on the exact setup, data reliability may be affected. Furthermore, details of the equipment choices are essential for attempts at replication.

#### Measurement conditions

It is important to include details about conditions at the time at which measurements are made. These include details of exogenous factors such as temperature, oxygenation, lighting and any sensory stimulation, such as from visual interactions with experimenters or with conspecifics in adjacent respirometers. Such factors may directly affect rates of animal oxygen uptake (Claireaux and Chabot, 2016; Clarke and Johnston, 1999; Nadler et al., 2016). Other important conditions include endogenous factors, such as the feeding state of the experimental animal and their adjustment period to experimental conditions; these aspects can also influence oxygen uptake (Chabot et al., 2016).

#### Background respiration

Over prolonged periods, throughout the alternating closed and flush phases, microbes may proliferate on the surfaces of respirometry systems. The magnitude of background microbial respiration can therefore be substantial and must be quantified and corrected for. There are several different methods that can be used, and the exact approach must be carefully documented.

#### Measurement of SMR (or RMR)

There are several methodological details that are specific to the measurement of either SMR or RMR (Box 1). As for the choice of equipment and setup, there are a range of possibilities; experimental details that can vary across studies must be clearly communicated. After data are collected, there are various ways to calculate values for SMR and RMR (Box 1). The trait value may be affected by the specific method chosen, and some approaches may even be inappropriate for the experimental conditions used or the species under study.

#### Measurement of MMR

As for the measurement of SMR and RMR, there are a variety of specific details that are unique to the measurement and processing of data for MMR (Box 1), most of which concern how increased rates of oxygen uptake are achieved. These details must be recorded to allow for accurate evaluation of the results and replicability of the experiment.

#### Data handling and analysis

There are several additional factors regarding basic data handling and processing that may affect final values or statistical analyses and that must, therefore, be provided to ensure that the data are interpretable and replicable.

## Survey of the existing literature

Following the development of the checklist introduced above, we conducted a quantitative analysis of previous reporting of the checklist criteria among studies using aquatic intermittent-flow respirometry in fishes. Our aim was to highlight specific areas in which reporting of methodological details can be improved. For details of how this literature survey was conducted, please see Appendix 1.

**Fig. 2.**
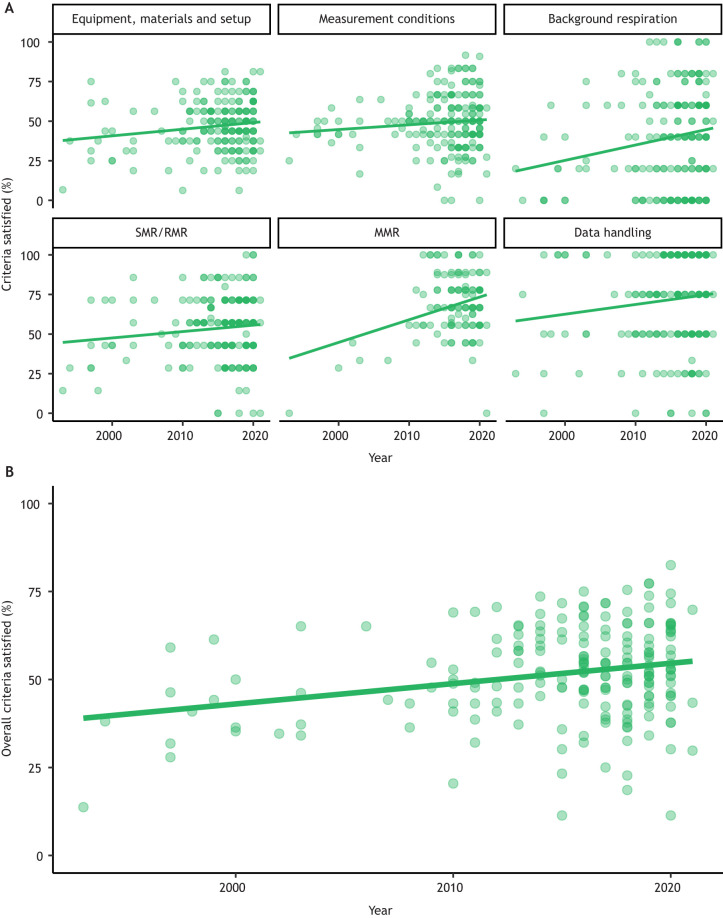
**The percentage of criteria listed in Table 1 that were satisfied in the surveyed papers.** Each point represents one paper; solid lines are linear regressions with publication year on the *x*-axis (see Table S2 for model summary and parameter estimates). Points are partially transparent and so darker shades of green indicate greater numbers of overlapping data points. (A) Criteria sub-divided according to category. (B) Overall percentage of criteria satisfied. The number of studies in each panel is *n*=202, except for the panel for MMR (many papers did not contain data for MMR, see Appendix 1), where *n*=123. Regression equations and *P*-values for effect of year are as follows: Equipment, materials and setup: *y*=−797.23*x*+0.419(year), *P*=0.0232; Measurement conditions: *y*=−551.42*x*+0.298(year), *P*=0.154; Background respiration: *y*=−1916.42*x*+0.971(year), *P*=0.00867; SMR/RMR: *y*=−758.29*x*+0.403(year), *P*=0.098; MMR: *y*=−2819.76*x*+1.43(year), *P*=0.0007; Data handling: *y*=−1165.27*x*+0.614(year), *P*=0.079; Overall: *y*=−1113.06*x*+0.578(year), *P*=0.0003.

**Fig. 3.**
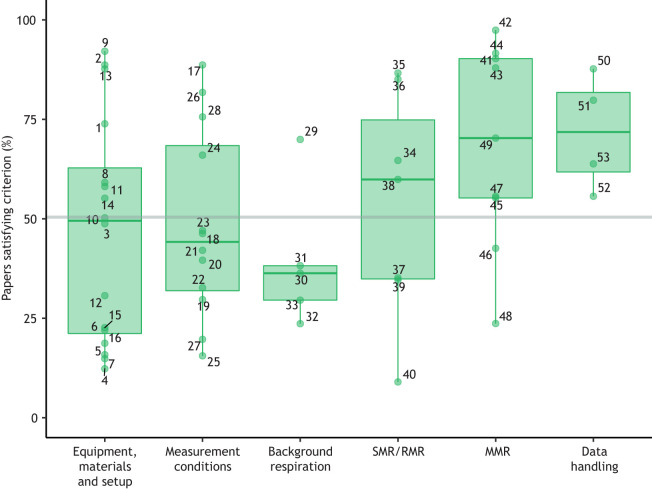
**The percentage of papers that referred to the specific criteria listed in Table 1.** Each point represents one criterion; numbered labels correspond to criteria numbering in Table 1. Criteria for each category were scored across *n*=202 studies, except for the MMR category, where *n*=123 (several papers did not include data for MMR, see Appendix 1). The grey line is the overall average across all criteria. Boxplot lower and upper hinges represent the 25th and 75th percentiles, respectively; the horizontal line within the box represents the median; the length of whiskers represents the range of data points between either the upper or lower hinge and 1.5× the difference between the 25th and 75th percentiles.

Our analysis of 202 published papers (with *n*=123 of these including data for MMR in addition to SMR or RMR), from 1993–2021, shows that reporting of methods has been relatively poor and inconsistent, including in our own published articles. Reporting showed a slight negative correlation with journal impact factor (Clarivate Analytics 2020, where available for each journal; GLMM, *P*=0.031), but there was tremendous variation around this relationship, indicating that problems with reporting persist throughout the published literature (Fig. S2; Table S2). There was some evidence of an improvement in reporting over the last few decades (Fig. 2; GLMM, effect of year, *P*=0.001), but the reporting of methods for intermittent-flow respirometry generally remains inadequate, with extreme variation in reporting quality among recent studies (Fig. 2). Although specific papers often scored highly within a particular category, all papers failed to report several criteria across categories. There was wide variation in reporting frequency of criteria within categories, with some specific criteria being consistently under-reported (Table 1; Fig. 3). The lack of consistency across studies is undoubtedly due to the lack of any established guidelines for reporting the methodological details of intermittent-flow respirometry.

In addressing specific methodological details, it is important for researchers to be as clear and explicit as possible, to eliminate any chance of misinterpretation. Another common problem is that articles often refer to multiple prior studies for methodological details, but these references would contain inconsistent or contradictory information. The use of inaccurate or vague phrasing can also cause confusion, misunderstanding of what methods were performed and, potentially, the spread of incorrect information and terminology. Of course, although the use of a checklist for methodological details should improve the reporting of methods for intermittent-flow respirometry, it is ultimately dependent upon researchers to use clear and unambiguous language when describing methods. Our findings on the reporting of various aspects of respirometry measurements across the literature are discussed in more detail below.

### 

#### Equipment, materials and setup

In our survey, only 71% of articles clearly specified the body mass of animals at the time of respirometry, as opposed to upon arrival at the lab or during holding conditions. Body size affects both minimum and maximum metabolic rates (Jerde et al., 2019; Killen et al., 2016) and temporal variation in body mass will affect metabolic rate estimation. This information is also required when assessing the ratio between respirometer volume and animal mass, a measure that was only explicitly provided in 12% of studies (criterion 4).

There were several other criteria that were consistently under-reported. For example, only 48% of papers surveyed provided any mention of whether mixing was performed inside the respirometers and how it was accomplished (criterion 3). Proper mixing of the water in respirometers is critical to homogenise oxygen concentrations throughout chambers, and accurately measuring oxygen uptake is simply not possible without effective mixing. Criteria relating to mixing circuit tubing (criteria 4, 5, 6 and 7) were only mentioned in 12–21% of papers, despite this being a key component of intermittent-flow respirometry methods that use an external mixing circuit (Rodgers et al., 2016; Svendsen et al., 2016a). In some respirometer designs, mixing may be achieved by using an impellor or stir-bar, but in situations where an external pump is used for mixing, any tubing used in a mixing circuit needs to be as clean as possible, as short as possible and made of relatively gas-impermeable material. Any respiration from microbes adhering to the surface, or gas exchange across the tubing, could have confounding effects that need to be corrected for or avoided. Moreover, the volume of the mixing circuit must be included in the calculation of respirometer volume and, therefore, animal oxygen uptake rate (criterion 7).

#### Measurement conditions

Numerous criteria pertaining to conditions during measurement were reported infrequently. Temperature or photoperiod during holding conditions were often given without explicit reference to conditions during respirometry, or how temperature conditions were maintained. Metabolic rates of ectothermic animals are profoundly influenced by temperature (Clarke and Johnston, 1999; Schulte, 2015) and photoperiod may affect animal oxygen uptake (Biswas and Takeuchi, 2002); temperature and photoperiod may also interact so that diurnal patterns in oxygen uptake are different at different temperatures (Speers-Roesch et al., 2018). Although many studies measure multiple animals simultaneously during respirometry, with each animal within its own chamber, only 15% of studies mention whether the animals were visually shielded from each other (criterion 25). This could have various impacts on activity and metabolic rates that could differ among species, depending on their level of sociability or aggression (Killen et al., 2014; Nadler et al., 2016; Ros et al., 2006). Only 19% of papers reported the total time taken to measure all animals in a study, from the start of the study to the end of the study (criterion 27). This criterion may be especially important for studies with large sample sizes, leading to overlap with breeding seasons or significant changes in mass of small, rapidly growing animals. Only 31% of studies mentioned the lowest water oxygen concentration that animals were exposed to during respirometer closed phases (criterion 22). If oxygen depletion by the animal actually causes hypoxia during closed phases, this may cause repeated reliance upon anaerobic pathways to meet energy requirements of metabolism, which would then interfere with estimates of metabolic rate that use oxygen uptake as a proxy (Snyder et al., 2016). Additionally, repeated hypoxia may elicit an endocrine stress response or stimulate swimming activity and increase ventilation frequency, also affecting metabolism and rates of oxygen uptake due to physical activity (Aboagye and Allen, 2014; Killen et al., 2012).

#### Background respiration

Overall, criteria associated with measuring background microbial (e.g. bacterial) oxygen uptake were the most inconsistently reported among studies (GLMM, effect of category, *P*<0.0001). In fact, more than 30% of papers surveyed did not mention whether any form of background microbial respiration measurement was performed or accounted for. This is a critical oversight because the amount of background respiration and the exact way it is measured, or incorrectly accounting for rates of background oxygen uptake, can greatly impact estimates of animal metabolic rates (Rodgers et al., 2016; Svendsen et al., 2016a).

A large proportion of remaining papers failed to describe how background respiration was controlled (e.g. by cleaning of respirometry chambers and setup), precisely how it was measured and accounted for, or the proportion of animal metabolism that it represented. Simply measuring background respiration prior to respirometry trials does not account for how it might increase with time of residence of animals in a respirometer. Background respiration should either be measured in parallel with animal respiration, using empty chambers, or should be measured at the start and finish of a trial, with a model decided upon for how it may have changed (increased) over time. In fact, parallel measurements of background respiration in empty chambers should ideally be combined with measurements at the start and finish of a trial in all chambers, as the temporal development of microbial respiration in chambers with animals may differ from that without animals. This is especially likely in studies where animals have been fed in the respirometers or before being placed in respirometers (e.g. studies that estimate the metabolic costs incurred during digestion and assimilation of nutrients, so called ‘specific-dynamic action’; McLean et al., 2018). Without appropriate details on background respiration, it is extremely difficult to assess data validity. This is, therefore, a methodological element that researchers must perform properly and report clearly.

### Estimating SMR/RMR and MMR

There are several criteria unique to the estimation of either SMR/RMR or MMR that were often not reported. Regarding SMR/RMR, the total number of oxygen uptake rate measurements (i.e. number of closed phases) used in the derivation of the metabolic rate estimate (criterion 37) was reported in only 34% of studies. Methods for statistically estimating SMR, for example, including the use of quantiles or frequency distributions, require a large number of repeated measures, and so the total number of slopes used in their derivation should be provided, as should any slopes that were disregarded during acclimation to the respirometer or periods of increased activity (criterion 38; including whether such periods were included in quantile- or frequency distribution-based methods of calculating SMR; Chabot et al., 2016). Note that there are excellent R packages available e.g. *respR* (https://github.com/januarharianto/respR; Harianto et al., 2019) and *FishResp* (https://CRAN.R-project.org/package=FishResp; Morozov et al., 2020) that are specifically designed for calculation of oxygen uptake rates using data obtained through intermittent-flow respirometry. These packages include various options for estimation of SMR, RMR and MMR, as well as corrections for background respiration. Wider use of these packages would help facilitate replication and reproducibility.

Although reporting for MMR was relatively good when compared with the other criteria categories (appearing in a mean of 61% of papers across criteria), there were still important details that were often neglected. For example, when measuring oxygen uptake immediately after exhaustion, many studies did not report whether the animal was exposed to air before placement in the chamber (criterion 45). The time taken to initiate measurements of oxygen uptake (e.g. in seconds, after the cessation of exercise) was also often not provided (criterion 46). Finally, only 21% of papers described the specific method of slope estimation for determining MMR. This is important because data processing procedures can bias estimates of MMR (Zhang et al., 2020), including the duration of the slope used to estimate MMR and the specific method of determining the maximum rate of oxygen uptake during recovery after exercise (criterion 48). Our survey revealed that both criteria were relatively underreported, but given emerging awareness of their importance, it is vital that authors provide these details going forward.

### Data handling

Additional basic information about data processing was also frequently under-reported. For example, while 85% of papers clearly reported study sample size, several did not provide this fundamental information or did so in a way that was unclear (i.e. provided total animals used in study but not specific treatments). Only 54% of papers specifically mentioned that animal volume was subtracted from total respirometer volume, a step in data processing that is required to obtain accurate rates of oxygen uptake. Finally, only 62% of papers explicitly stated whether they used any form of body size adjustment or correction for rates of oxygen uptake in analyses or described their methods for doing so. Due to the strong correlation between body size and whole-animal metabolic rate (and corresponding oxygen uptake), this information is key for ensuring data interpretability.

## Conclusions

As discussed above, the reporting of methods for intermittent-flow respirometry has generally been inconsistent and insufficient. However, we hope the development of guidelines and the availability of a reporting checklist will facilitate systematically clear and accurate reporting of methods. Reporting needs to be improved for all the general areas we examined and for specific criteria, but especially when considering measurement of background respiration and details of the mixing circuit. Although we suggest that intermittent-flow respirometry should be the method of choice whenever possible, elements of the checklist related to methodological details, measuring conditions and background respiration are also relevant to the general use of other forms of respirometry to estimate metabolic rate in aquatic animals.

As authors that frequently use intermittent-flow respirometry, we appreciate the challenges in reporting the numerous details required for adequate replication and interpretation of data collected using this technique. We acknowledge that our own work has been prone to the same reporting deficiencies we have described in this Commentary and, indeed, many of our own papers are contained within our literature survey. We suggest that authors use our downloadable checklist (Table S1) to concisely address all the criteria outlined in the current paper and that authors make a completed version of this form available as a supplementary table in future published papers. This checklist can also be used to help carefully plan important details when designing setups for the collection of oxygen uptake data.

It is more important than ever to ensure the collection and reporting of reliable and replicable data, as metabolic rates are increasingly becoming a focus for understanding the ability of animals to cope with environmental and climate change. Accurate reporting of methodologies is particularly important in cases where data may be used to inform conservation efforts. The availability of a checklist of important methodological details should also be useful to new researchers entering this rapidly developing field, and we hope that our checklist will be a valuable resource to both new and experienced researchers in this area.

## Supplementary Material

Supplementary information

## Appendix 1

### Methods for the literature survey and scoring of criteria

#### Literature survey and criteria scoring

Focussing on studies with fish, we performed a survey of the literature to determine variation in the reporting of methods and the extent to which various criteria are (or are not) reported. Using Web of Science and Google Scholar in April 2021, we used the topic search terms: (1) ‘fish AND respirometry AND intermittent’; and (2) ‘[fish AND (“standard metabolic rate” OR “resting metabolic rate” OR “routine metabolic rate”) AND “maxim* metabolic rate”]’. This survey was not meant to be exhaustive but was meant to be representative of the methodological reporting across research using fish intermittent-flow respirometry as a whole. Articles were excluded from further analysis if they were review articles, meta-analyses or any other study that did not estimate metabolic rates of fish using intermittent-flow respirometry. Studies that estimated metabolic rates while the animal was in a swim-tunnel were also not included in the survey (i.e. we only included studies where measurements of oxygen uptake were performed in static respirometry chambers). In total, 202 studies from 71 journals were assessed from between the years 1993 and 2021 (data are available at Mendeley Data: https://doi.org/10.17632/fky5n2nt9x.2; and are also included with this submission). This consisted of 123 studies that measured both SMR (or RMR) and MMR, and 79 studies that measured or SMR (or RMR) only, without MMR.

Each study was scored for whether they satisfied each criterion in the checklist. Studies were awarded a point for a given criterion if they gave a clear, unambiguous description of that methodological detail, without the need for reader assumptions or calculations. Importantly, scores were not based on the quality of a methodology itself – they were simply based on whether a given detail was provided. For example, if a paper had stated that the respirometer was made of Swiss cheese, the criterion ‘provide material of respirometer’ (criterion 8; Table 1) would be considered satisfied and a point would be awarded, without judgement of whether Swiss cheese is an appropriate material for respirometer construction. Methodological details for specific criteria were considered present if they were provided in the main article text, figures, tables or supplementary material, or in references to previously published work. When there were references to multiple prior studies for a given criterion, a point was not given if those prior sources provided inconsistent or contradictory descriptions. In some cases, the absence of a specific criterion made it impossible to assess other associated criteria, in which case a value of NA was assigned to criteria that were unable to be scored, and those instances were not included in calculating the mean score for that paper or in calculating the mean prevalence of that criteria across papers. Although most studies were evaluated by one scorer, 16 studies were initially evaluated by two scorers each, ensuring consistency across scorers and allowing refinement of criteria phrasing to minimise ambiguity. For each article, we also recorded the title, year of publication and journal (paper titles have been anonymised in the data file at Mendeley Data: https://doi.org/10.17632/fky5n2nt9x.3).

#### Statistical analysis

A generalised linear mixed model (GLMM) with a binomial distribution (logit link) was constructed to examine factors affecting methods reporting across published papers. The score for each criterion per paper (0 or 1) was used as the response variable, and criteria category, scaled year, scaled journal impact factor and all interactions among these variables were initially included as explanatory variables. Paper ID (coded anonymously by title) and scorer were included as random effects. Non-significant interactions were dropped sequentially and the model re-run. All analyses were conducted using R v. 4.0.3 (https://www.r-project.org/) using the function glmm in package *lme4* (https://CRAN.R-project.org/package=lme4; Bates et al., 2015). All R scripts are available at Mendeley Data: https://doi.org/10.17632/fky5n2nt9x.3.
