# Supplementary material for: Guidelines for reporting methods to estimate metabolic rates by aquatic intermittent-flow respirometry
Source: J Exp Biol. 2021 Sep 14;224(18):jeb242522. doi: 10.1242/jeb.242522 (PMC8467026; doi:10.1242/jeb.242522)
Supplement: Supplementary information [file jexbio-224-242522-s1.pdf]

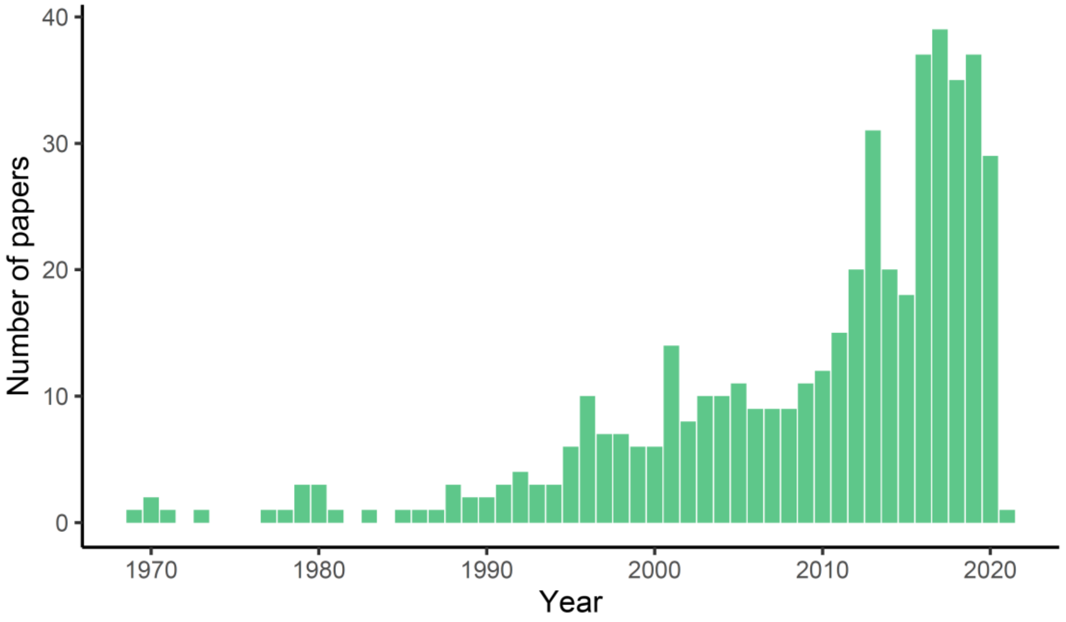

Fig. S1. Research in aquatic respirometry has increased steeply over the last several decades. The graph shows the number of papers per year, returned by the topic search [aquatic AND respirometry] on Web of Science (February 2021).

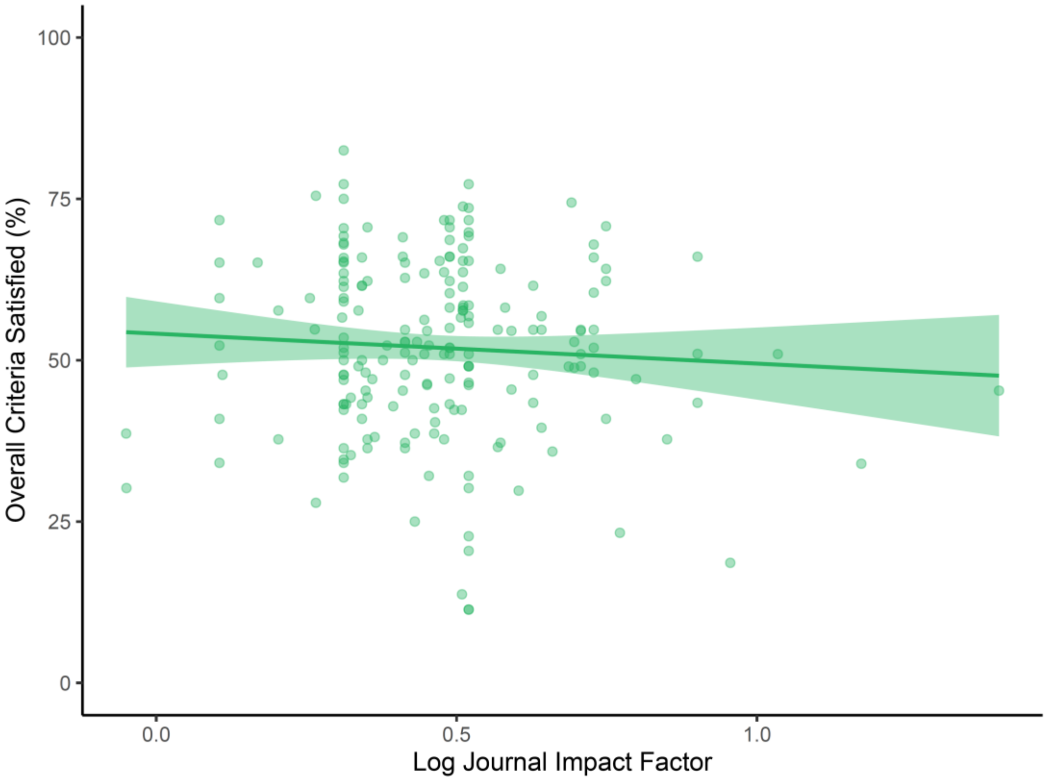

Fig. S2. The percentage of criteria listed in Table 1 that were satisfied in papers, in relation to the impact factor of the journal each paper was published in. Each point represents one paper (n = 202); the solid line is a linear regression with log journal impact factor on the x-axis and the shaded area represents 95% CIs (see Table S2 for model summary and parameter estimates). Darker shades of green indicate greater numbers of overlapping data points.

Table S1. The checklist of 53 essential criteria for the reporting of methods for aquatic intermittent-flow respirometry. This table can be copied by authors and included as supplemental information when submitting manuscripts and publishing papers. This will facilitate clear and concise means for reporting all important methodological details, with the article main text being used to provide additional information and context.

Click here to download Table S1

Table S2. Summary for the generalised linear mixed model with a binomial distribution (logit link), constructed to examine factors affecting methods reporting across published papers. The score for each criterion per paper (0 or 1) was used as the response variable, and criteria category, year, journal impact factor, and all interactions among these variables were initially included as explanatory variables. Year and impact factor were scaled. Paper ID (coded anonymously by title) and scorer were included as random effects. For the term 'category', background respiration is the reference level.

term	estimate	s.e.	z	p
intercept	-0.432	0.093	-4.627	< 0.0001
year	0.130	0.041	3.186	0.0014
impact factor	-0.083	0.038	-2.155	0.031
category				
Equipment and Setup	0.322	0.078	4.127	< 0.0001
Measurement conditions	0.435	0.081	5.368	< 0.0001
SMR/RMR	0.610	0.089	6.870	< 0.0001
MMR	1.212	0.099	12.129	< 0.0001
Data handling	1.398	0.108	13.100	< 0.0001

Journal of Experimental Biology • Supplementary information
